# How do treatments for chronic fatigue syndrome work? Exploration of instrumental variable methods for mediation analysis in PACE – a randomised controlled trial of adaptive pacing therapy, cognitive behaviour therapy, graded exercise therapy, and specialist medical care

**DOI:** 10.1186/1745-6215-12-S1-A144

**Published:** 2011-12-13

**Authors:** Kimberley Goldsmith, Trudie Chalder, Peter White, Michael Sharpe, Andrew Pickles

**Affiliations:** 1 Institute of Psychiatry, King's College London, DeCrespigny Park, London, SE5 8AF, UK; 2 Wolfson Institute of Preventative Medicine, Queen Mary, University of London, EC1M 6BQ, UK; 3 Department of Psychiatry, University of Oxford, OX3 7JX , UK

## Objectives

### Background

Chronic fatigue syndrome (CFS) is characterised by chronic disabling fatigue. The PACE trial compared four treatments for CFS and found that for therapies added to specialist medical care (SMC), cognitive behaviour therapy (CBT) and graded exercise therapy (GET) were more effective than adaptive pacing therapy (APT) and SMC alone in improving physical function and fatigue. What are the mechanisms of these treatments? CBT and GET may affect outcomes through thought processes and behaviours (mediators). Traditional Baron, Judd and Kenny (BJK) methods for estimating mediation effects can be subject to bias; instrumental variable methods (IV) can address this problem. The aims were:

To explore potential IVs for causal analysis of mediation in PACE.

To compare IV estimates to those obtained using BJK methods, which are unbiased only under restrictive assumptions such as no unmeasured confounding.

## Methods

Two treatment arms were compared at a time. BJK methods were applied using three ordinary least squares (OLS) regression models. IV methods were applied by compiling a list of baseline variables that could act as IVs in interaction terms with treatment arm and then assessing these using OLS with the mid-treatment measurement of the putative mediator as the outcome. Instrument strength was assessed using the R^2^ change between models with main effects only and with the interaction term. Two-stages least squares regression (2SLS) was used to estimate effects in the presence of IVs. Collective instrument strength was assessed using an F test and partial R^2^.

## Results

The IVs were weak, with a maximum R^2^ change of 0.03. The five strongest IVs were therefore used in the 2SLS in each case. There was modest mediation of CBT and GET effects (approximately 20% of the total effect). The IV-derived estimators were somewhat different in magnitude than the BJK estimators and were less precise. There is scope for modelling a common effect of mediators on outcomes across trial arms.

## Conclusions

There was evidence for modest mediation of CBT and GET effects. Potential IVs for the study of PACE treatment mechanisms can be found, however, these were weak. Combining trial arms may allow for more efficient analysis using IVs.

